# Dental professionals' experience with and handling of suspicion of child maltreatment in a small‐scale society, the Faroe Islands

**DOI:** 10.1002/cre2.164

**Published:** 2019-01-31

**Authors:** Unn Jakobsen, Anna Sofia Fjallheim, Hannes Gislason, Eina Gudmundsen, Sven Poulsen, Dorte Haubek

**Affiliations:** ^1^ Municipal Dental Service for Children and Adolescents, Klaksvík Faroe Islands; ^2^ University of the Faroe Islands, Faculty of Health Sciences, Centre of Health Science, Sjólon Vestara Bryggja Tórshavn Faroe Islands; ^3^ University of the Faroe Islands, The Faculty of Science and Technology, Sjólon Vestara Bryggja Tórshavn Faroe Islands; ^4^ Municipal Dental Service for Children and Adolescents, Department of Orthodontics, Tórshavn Faroe Islands; ^5^ Section for Paediatric Dentistry, Department of Dentistry and Oral Health, Health Aarhus University Aarhus Denmark

**Keywords:** Child maltreatment, Child protection, Small‐scale society, Dental professionals

## Abstract

The aims of the present study were to describe how frequently dental professionals in a small‐scale society like the Faroese Islands, experience suspicion on child maltreatment, and how they handle their suspicion. Furthermore, we wanted to investigate the hypothesis that the special interpersonal characteristics of small‐scale societies like the Faroese, influence how dental professionals handle suspicion of child maltreatment compared to how their colleagues in larger societies handled such suspicion.

The design of our study was cross‐sectional using a non‐probability purposive sampling method. A translated and slightly modified version of the Danish questionnaire regarding suspicion on child maltreatment was sent to all 71 dental professionals (44 dentists and 27 dental hygienists) in the Faroe Islands. 51 (72%) returned a valid questionnaire. Of these, 61% experienced suspicion of child maltreatment at some point in their career, 33% within the last 6 months, and 10% percent were certain of child maltreatment during the last six months. Of those respondents who had experienced suspicion at some point of their career, 39% had reported their suspicion. The main reasons for withholding a suspicion were: uncertainty as to whether the suspicion was reliable, fear of the consequences for the child, and lack of procedural knowledge. Faroese dental professionals suspected child maltreatment much more frequently (61%) than their Danish (38%), Scottish (29%), and Croatian colleagues (26%) did. Child maltreatment raises concern among the Faroese dental professionals more frequently than among their colleagues in larger societies. They also seem to report their concern more frequently than their Scottish colleagues do. Thus, the present study indicates that the social structure in small‐scale societies may affect dental professionals' suspicions, and handling of child maltreatment.

1

**What this paper adds**

- Information regarding dental professionals' handling of suspicion of child maltreatment
- A hypothesis that dental professionals' handling of suspicion of child maltreatment seems to be somewhat different in the small‐scale societies

**Why this paper is important for pediatric dentists**

- It includes social structures in the understanding of dental professionals' handling of suspicion of child maltreatment
- Pediatric dentists should be familiar with as many aspects of child maltreatment as possible

## 2 INTRODUCTION

Child maltreatment includes all forms of physical and emotional ill‐treatment, sexual abuse, neglect, and exploitation that results in actual or potential harm to the child's health, development or dignity (World Health Organisation, n.d.). The exact proportion of children being affected can be difficult to determine due to the lack of notification of all cases. However, a recent Danish population survey of 24 year‐olds found that 3.0% had experienced physical neglect, 5.2% had experienced emotional abuse, 5.4% had experienced physical abuse, and 3.4% had experienced sexual abuse before the age of 12 years (Christoffersen, Armour, Lasgaard, Andersen, & Elklit, 2013). Furthermore, a literature review of studies conducted in the Nordic countries (but not including The Faroese Islands) on sexual abuse (contact abuse or penetrating abuse) found prevalence rates of 3–23% for boys and 11–36% for girls (Kloppen, Haugland, Svedin, Mæhle, & Breivik, 2016). Unfortunately, prevalence rates for child maltreatment at the Faroese Islands are not available from the literature. As child maltreatment has serious consequences (Gilbert et al., 2009), it deserves the attention of all health professionals meeting the child, including dental professionals.

Dentists and dental hygienists are in a unique position to notice symptoms of child maltreatment, as 50 to 70% of all abused children have visible injuries on the head or in the neck region (Cairns, Mok, & Welbury, 2005a; Jesse, 1995; Welbury & Murphy, 1998). However, previous studies indicate a lack of knowledge in the professional dental teams on how to handle suspicions, including barriers and lack of knowledge about procedures for reporting such suspicions (Brattabo, Iversen, Astrom, & Bjørknes, 2016; Cairns, Mok, & Welbury, 2005b; Cukovic‐Bagic et al., 2015; Drigeard, Nicolas, Hansjacob, & Roger‐Leroi, 2012; Hashim & Al‐Ani, 2013; Kaur et al., 2016; Laud, Gizani, Maragkou, Welbury, & Papagiannoulis, 2013; Mogaddam, Kamal, Merdad, & Alamoudi, 2016; Owais, Qudeimat, & Qodceih, 2009; Uldum, Christensen, Welbury, & Poulsen, 2010; Uldum, Christensen, Welbury, & Haubek, 2017). The available reports on this topic are all conducted in large‐scale societies, but one of these (Brattabo et al., 2016) showed that public dental health personnel working in smaller municipalities (≤ 10,000 inhabitants) reported suspicion of child maltreatment less frequently than dental public health personnel working in larger municipalities. However, to our knowledge, no studies have yet examined the handling of suspicions among dental professionals in small‐scale societies, which in many aspects differ from large‐scale societies. The Faroe Islands is such a small‐scale society with a population of just above 50.000 in year 2018. It is a part of the Danish Kingdom, but has autonomy in several governmental matters, including health services. The Faroese government provides diagnostic and preventive dental care and treatment to all children and youths from 0 to 18 years of age free of charge. The service is provided either from public clinics or private practices having a contract with the municipality. Practically, all children and young adults are regularly seen and treated by dental professionals. Dental professionals in the Faroe Islands are obliged to report any suspicion of abuse or neglect against a minor to the Child Welfare Services, which, in case of sexual abuse or violence, collaborate with the Health Services and the Police. Since 2013, the person submitting the report to the Child Welfare Services has to be named, and the obligation to report overrides other legal regulations to ensure confidentiality. In 2014, 743 cases were reported to the Child Welfare Services. Among these, 20 proved to be violence cases, 7 for violence, 10 sexual abuse cases, and three both sexual abuse cases and violence cases (http://www.bvs.fo/barnaverndarstovan/hagtoel; http://www.bvs.fo/barnahusið/fragreiðingar).

A defining characteristic of small‐scale societies is the multiplex relational structure (Gluckman, 1955). People relate to each other in myriad of connections that coincide and overlap extensively. This makes anonymity near impossible (Benedict, 1966), which is also found to be the case in other Faroese studies (Gaffin, 1996). In addition, relationships tend to be particularistic rather than universalistic, which is to say that people relate to one another on a personal level over any professional connection. This can complicate one's professional distance (Benedict, 1967), often a necessity in reporting a case of child maltreatment. Particularistic relationships also heavily favor avoiding open conflicts (Benedict, 1967). Therefore, it could be hypothesized that very close relational ties between people in very small community, like the Faroe Islands, might affect the professionals' handling of suspicions of child maltreatment. The aims of the present study were to describe how frequently dentists and dental hygienists in the Faroe Islands experience suspicion of child maltreatment, including to what extent they report their suspicions, to whom they report their suspicions, and the barriers related to such situations. Furthermore, we wanted to investigate the hypothesis that the special interpersonal characteristics of small‐scale societies like the Faroese, influence how dental professionals handle suspicion of child maltreatment compared to how their colleagues in larger societies handled such suspicion.

## 3 MATERIALS AND METHODS

The study was conducted as a cross‐sectional investigation. The sampling method was a non‐probability purposive sampling method, including the total population of dentists and dental hygienists in the Faroe Islands.

### 3.1 Questionnaire

The data reported in the present study were obtained by the use of the Danish version of the original questionnaire, designed by Cairns and coworkers (Cairns et al., 2005b), which has previously been used in two studies carried out in Denmark (Uldum et al., 2010; Uldum et al., 2017). The questionnaire was translated from Danish to Faroese by a qualified dentist with a mastery of the Faroese language. Subsequently, the questionnaire was then back‐translated into Danish by two translators. Disagreements were discussed and some adjustments were made accordingly. Afterwards, the questionnaire was tested by five pedagogues to ensure that the questions were understandable and unambiguous. A copy of the questionnaire can be obtained from the first author.

As the Faroe Islands is a relatively small community, it has been necessary to modify certain questions to ensure anonymity. The questions regarding the place of education and numbers of own children of the participants were excluded. The age of the participants, which in the Danish questionnaire had to be answered in years, was categorized into three age groups: 35 years or younger, 36 to 50 years, and above 50 years. The question regarding the size of the participant's local municipality was categorized into two categories: municipalities with 2000 people or more (Argir, Hoyvík, Klaksvík, and Tórshavn districts), and municipalities with less than 2000 people (all other villages in the Faroe Islands).

Gallup Føroyar was responsible for the data collection of the questionnaire with 32 questions. The data were stored in an Excel file containing one row for each respondent with 45 columns of answers, since answers to multiple‐choice questions were stored in multiple columns.

### 3.2 Study population

The target population consisted of all 71 dental professionals (44 dentists and 27 dental hygienists) in the Faroese Islands. Retired affiliates were excluded from the study population. Dentists in private practice were included as they may still be consulted by children in acute need for emergency care.

The lists of names and email addresses to whom the questionnaires were sent, were obtained from Tannlæknafelagnum [The Society of Dentists] and Felagið Føroyskir Tannrøktarir [The Society of Faroese Dental Hygienists], The Faroe Islands. The questionnaire was sent to the Faroese dentists and dental hygienists digitally.

An explanatory note was written and sent to every participant January 18^th^ 2016, and the digital questionnaire, with hyperlink to a personal login and password, was sent January 20^th^ 2016. The initial deadline for answering was February 7^th^. Two reminders were sent after one and two weeks. Due to a low number of completed questionnaires, the deadline for participation was extended, and the data collection finalized February 15^th^ 2016.

### 3.3 Ethics

The study was approved by the Faroese Data Protection Agency. No further formal approval was needed for the study, according to the Faroese Data Protection Agency.

### 3.4 Statistical methods

The questionnaire data was imported for data wrangling and analysis with RStudio and the *tidyverse* R‐package (Wickham & Grolemund, 2016). Summary statistics of answers (counts and percentages) were created and presented in tables and plots. The respondents' suspicion of child maltreatment were compared with studies from Denmark (Uldum et al., 2010), Scotland (Cairns et al., 2005b) and Croatia(Cukovic‐Bagic et al., 2015), in which the same questionnaire had been used, on the following variables: “ever had suspicion”, and “had suspicion within the last six months”; with Denmark and Croatia on the variable “sure about suspicion within the last six months”; and with Denmark and Scotland on the variable “reported suspicion at some point of their career”. The proportions (%) for the 51 answers in the Faroe Islands are assumed to approximate the true proportion (*H*_*0*_) in the total (*n* = 71) dental staff population that was sampled. The proportions (%) in the other countries are compared to *H*_*0*_ in the Faroe Islands and to a worst case non‐response biased hypothesis *H*_*0*_ (bias), where we assumed all the non‐respondent participants in Faroe Islands to have no suspicion. The exact binomial test was used to compute the sample proportions (%, rounded in table), the 95%‐confidence intervals *CI,* and the *p*‐values. A *p*‐value <0.05 rejects equality of proportions and *H*_*0*_ or *H*_*0*_ (bias).

## 4 RESULTS

In total, 51 (72%) of the 71 members of the target population returned a questionnaire with valid data. Of these, 33 were dentists and the remaining 18 were dental hygienists (Table 1). Nine (27%) of the 33 dentists, and 8 (44%) of the dental hygienists were employed in municipal dental service. In total, 18 did not answer and two did not complete the questionnaire correctly. The 51 respondents were fairly equally distributed according to employment (Table 1).

**Table 1:** Distribution of respondents according to age, profession, employment, and size of population in location where they work

Age	≤ 35 years	14 (28%)
	36–50 years	20 (39%)
	> 50 years	17 (33%)
Professional background	Dental hygienists	18 (35%)
	Dentists	33 (65%)
Employment	Municipal dental service	13 (26%)
	Private practice with children	16 (31%)
	Private practice without children	14 (28%)
	Other (including unemployment)	8 (16%)
Size of population in location where they work	< 2,000 persons	16 (31%)
	≥ 2,000 persons	35 (69%)

Thirty‐one (61%) of the respondents experienced suspicion of child maltreatment at some point during their career (i.e., ‘ever’), 17 (33%) during the last six months, and 5 (10%) were certain about their suspicion during the last six months. 39% (12/31) had given notice of their suspicion in at least one case during their career: 6 of 33 dentists (18%) versus 6 of 18 hygienists (33%), whereas 71% (22/31) did not notify anyone about their concern. It is worth noting that some respondents experienced suspicion in several cases, some of which they might have reported, while they in other cases had withheld their suspicion. Among 16 respondents working in smaller municipalities (less than 2000 inhabitants), 11 (69%) experienced suspicions of maltreatment. In larger municipalities (more than 2000 inhabitants), 20 (57%) out of 35 respondents experienced suspicions.

**Figure 1:**
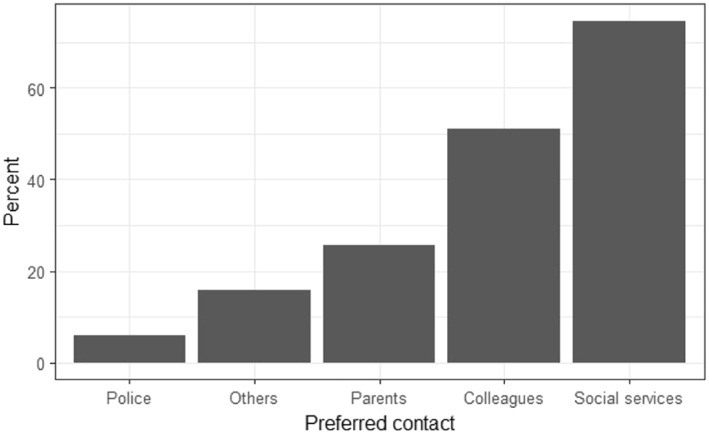
Respondents' distribution according to whom they would prefer to report their suspicion of child maltreatment

A multiple‐choice question allowing multiple answers about a hypothetical case of suspicion revealed that 38 of the 51 respondents would report their suspicions to the social services, 26 to their colleagues, and 13 to the parents (Figure 1). Only three would contact the police.

**Figure 2:**
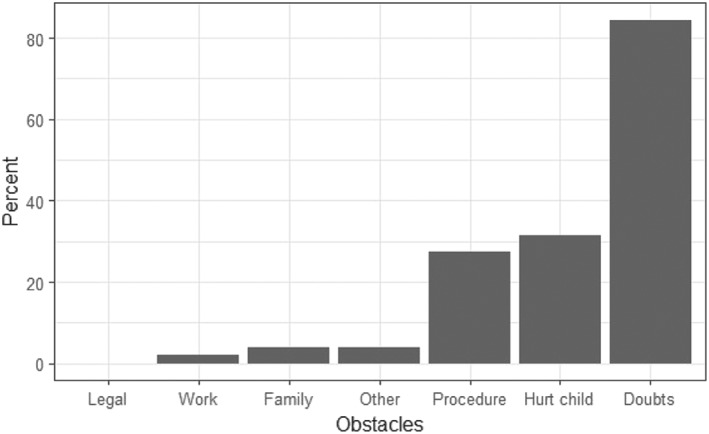
Respondents' distribution according to which barriers they would experience against reporting child maltreatment

The three main reasons for withholding a suspicion were (Figure 2): Uncertainty as to whether the suspicion was reliable (46 answers), fear of the consequences for the child (16 answers), and lack of procedural knowledge (14 answers). No respondents hesitated reporting their suspicion due to concern for having to attend court over the matter.

When asked, if they considered themselves sufficiently informed about the proper procedure to follow in case of suspicion of maltreatment, 46 of the 51 respondents (90%) responded dismissively. Ten (56%) of the dental hygienists had received training in management of child maltreatment as part of their undergraduate training compared to 12 (36%) of the dentists. Nine (27%) of the dentists and 4 (22%) of the dental hygienists had received training in this subject as continuing education. Thirty‐nine (76%) felt a need for further education in the area, and only 2 (4%) had received guidelines on how to handle suspicion on child maltreatment from the municipal authorities.

When comparing data from the present study to similar data from Denmark, Scotland and Croatia, in which the same questionnaire had been used, we found marked, statistical significant differences in dental professionals' management of suspicion of child maltreatment for most of the parameters (Table 2). The proportion that experienced suspicion about child maltreatment at one point in their professional life was considerable higher for Faroese dental professionals (61%) than for Danish, Scottish, and Croatian dental professionals (38%, 29%, and 26%, respectively). This difference was statistical significant, even under the worst case non‐response biased hypothesis. Similarly, the differences are large between the data regarding suspicion in the last six months from the Faroe Islands as compared to the data reported from Denmark, Scotland and Croatia (Table 2). Information on the proportion of dental professionals being sure about suspicion during the last six months were not available from the Scottish study. Concerning this matter, we found a proportion of 10% in the Faroese study and the corresponding proportions of 7% and 1% in the Danish and Croatian studies, respectively. Here, the difference between the Faroese (10%) and the Danish study (7%) is significant (*p* = 0.001 < 0.05), if we disregard the hypothetical non‐response bias *H*_*0*_ (bias) = 7% (*p* = 0.905). The proportions of reports of suspicion in the Faroese (39%) and the Danish study (34%, *CI*: 30–39%) are not significantly different (*p*‐value = 0.068), whereas the proportion in the Scottish study was considerably lower (8%, *CI*: 4–15%). There was no data concerning reports in the Croatian study.

**Table 2:** Comparison of Faroese dental professionals' suspicion and handling of child maltreatment compared to results from similar studies in Denmark (Uldum et al., 2010), Scotland (Cairns et al., 2005b), and Croatia (Cukovic‐Bagic et al., 2015)

Variable	Country	Yes	*n*	%	*CI*	*H*_*0*_	*p‐*value	*H*_*0*_ (bias)	*p‐*value
“ever had suspicion”	**Faroe Islands**	31	51	61	‐	**61**	‐	**44**	‐
	Denmark	433	1145	38	35–41	61	0.000	44	0.000
	Scotland	109	375	29	25–34	61	0.000	44	0.000
	Croatia	134	510	26	23–30	61	0.000	44	0.000
“had suspicion within last six months”	**Faroe Islands**	17	51	33	‐	**33**	‐	**24**	‐
	Denmark	146	1077	14	12–16	33	0.000	24	0.000
	Scotland	5	375	1	0–3	33	0.000	24	0.000
	Croatia	21	510	4	3–6	33	0.000	24	0.000
“sure about suspicion within last six months”	**Faroe Islands**	5	51	10	‐	**10**	‐	**7**	‐
	Denmark	74	1081	7	5–9	10	0.001	7	0.905
	Croatia	5	510	1	0–2	10	0.000	7	0.010
“reported suspicion at some point of their career”	**Faroe Islands**	12	31	39	‐	**39**	‐	**‐**	‐
	Denmark	149	433	34	30–39	39	0.068	‐	‐
	Scotland	9	109	8	4–15	39	0.000	‐	‐

For the Faroese respondents, the social services were their first choice to report their suspicion to, followed by colleagues as their second, whereas the Danish and Croatian respondents preferred colleagues as their first choice and social services as their second. Both the respondents in the Faroese and in the Danish study would discuss this issue with the parents/caregiver as a third choice, but the Danish respondents would have far more inquires to the caregiver than the Faroese respondents. In the Croatian study, referral to the parents was not an option.

Barriers experienced by the respondents in the present study were somewhat similar to those previously reported from Denmark and Croatia. However, in the Faroese sample, uncertainty was the most frequent reason for deciding not to report concern, followed by fear of consequences to the child. Thus, it is mainly the uncertainty that keeps Faroese respondents from reporting their suspicions. The respondents in the Danish study had in general experienced more barriers than the respondents in the Faroese study. Both in the Croatian study and the Danish study, some respondents hesitated reporting their suspicion due to concern for having to attend court over this matter compared to the Faroese study where no one saw this barrier as an obstacle to report.

## 5 DISCUSSION

To our knowledge, this is the first study to examine the handling of suspicion of child maltreatment by dental professionals in a small‐scale society like the Faroe Islands.

The response rate in the present study was higher than in the Danish study (Uldum et al., 2017) reported on in 2017 (67%), similar to the response rate found in the Danish study from 2010 (Uldum et al., 2010) (76%), and in the Scottish study (Cairns et al., 2005b) (75%), but lower than in the Croatian study (Cukovic‐Bagic et al., 2015) (93%). However, as non‐response sampling‐bias was one of our main concerns, and as such bias may seriously affect the study conclusions, we simulated the worst‐case non‐response bias (Table 2).

The non‐probability purposive sampling method was chosen for this study, as The Faroese Islands is a small‐scale country, and it was logistically possible to include all dental professionals in the sample. Drawing a random probability sample would have reduced the power of the study even more. However, the small sample size makes analysis of contrasts between subgroups difficult. Increasing the sample size by including, e.g., retired dental professionals was not an option, since we wanted to describe the present‐day situation.

Finally, the risk of recall bias should be considered, especially as some of the questions concerned the respondents' entire professional career. It could be argued that the risk of recall bias could have been reduced, if the respondents had been instructed only to respond on the basis of information from the children's dental records. However, it is doubtful if data on suspicion of child maltreatment have been recorded, as it is not part of the dental information routinely recorded in the child's record.

In accordance with theoretically and empirically‐derived properties of small‐scale societies, we found dental professionals' handling of suspicion of child maltreatment to be different in the Faroe Islands compared to large‐scale societies, like Denmark (Uldum et al., 2010; Uldum et al., 2017), Croatia (Cukovic‐Bagic et al., 2015) and Scotland (Cairns et al., 2005b). Especially, suspicion of child maltreatment was experienced much more frequently by Faroese dental professional than by their Danish, Scottish, and Croatian colleagues. This might be due to the low level of anonymity in Faroe Islands, affecting the general knowledge that people have about each other. Thus, dental professionals might have knowledge about their patients beyond the knowledge gained from the professional relationship. This Faroese sample showed a high degree of suspicion, but at the same time a high degree of uncertainty about the suspicions, which might be due to the characteristics of extensive knowledge people in general have about each other in small‐scale societies like the Faroe Islands. Especially knowledge, which is obtained through personal rather than professional relations, may result in an uncertainty related to the source of one's suspicion. This supports the hypothesis that social structures in a small‐scale society might affect the handling of suspicions.

The Faroese dentists and dental hygienists have the same educational background as their Danish colleagues, as almost all are educated from one of the two Danish universities. Therefore, the differences in suspicion might not be due to differences in the educational background.

Surprisingly, the Faroese respondents chose to report their suspicion to the social service above the other options, when asked about an imaginary case of suspicion. It could have been expected that their first choice would have been colleagues, as this was the case in both the Danish and the Croatian study. Considering both the high degree of uncertainty about the reliability of own suspicions and not least the avoidance of conflicts in small‐scale societies, it might have been more natural to turn to colleagues first in order to validate the suspicion before reporting it to the social services. The unexpected response might be due to the tendency to respond in accordance with what society favors to be the correct answer (Paulhus, 2002). In recent years, an increased awareness has been paid to the obligation to report suspicions of child maltreatment, and in particular the extended obligation of professionals. On the other hand, almost all respondents in this study would also need to discuss their concern with a colleague.

In the present study, the biggest barrier that Faroese dental professionals perceived for reporting child maltreatment was the uncertainty of their observations, which is similar to findings from the Danish study. In contrast to the Danish study, where the respondents perceived several other barriers, the Faroese dental professionals did not consider other barriers as pronounced. The uncertainty might be a sign that the dental professionals need further education on this subject, as the results of the present study also indicate. Another explanation can be the lack of local child protection guidelines. Only very few respondents had received such guidelines.

In conclusion, the present study indicates that dental professionals' in the small‐scale society of the Faroe Islands experience concern of child maltreatment more frequently than their colleagues in large‐scale societies, like Denmark, Croatia and Scotland. They also report their concern more frequently than their Scottish colleagues do. More studies, probably using also qualitative research methods, are needed to further our understanding.
